# BCG Signal Quality Assessment Based on Time-Series Imaging Methods

**DOI:** 10.3390/s23239382

**Published:** 2023-11-24

**Authors:** Sungtae Shin, Soonyoung Choi, Chaeyoung Kim, Azin Sadat Mousavi, Jin-Oh Hahn, Sehoon Jeong, Hyundoo Jeong

**Affiliations:** 1 Department of Mechanical Engineering, Dong-A University, Busan 49315, Republic of Korea; stshin@dau.ac.kr (S.S.); synike@naver.com (S.C.); 2 Institute for Digital Antiaging and Healthcare, Inje University, Gimhae 50834, Republic of Korea; chaeyoung0584@gmail.com; 3 Department of Mechanical Engineering, University of Maryland, College Park, MD 20742, USA; amousavi@umd.edu (A.S.M.); jhahn12@umd.edu (J.-O.H.); 4 Department of Healthcare Information Technology, Inje University, Gimhae 50834, Republic of Korea; 5 Paik Institute for Clinical Research, Inje University, Busan 50834, Republic of Korea; 6 Department of Mechatronics Engineering, Incheon National University, Incheon 22012, Republic of Korea

**Keywords:** ballistocardiogram, classification, convolutional neural network, signal quality assessment, time-series imaging

## Abstract

This paper describes a signal quality classification method for arm ballistocardiogram (BCG), which has the potential for non-invasive and continuous blood pressure measurement. An advantage of the BCG signal for wearable devices is that it can easily be measured using accelerometers. However, the BCG signal is also susceptible to noise caused by motion artifacts. This distortion leads to errors in blood pressure estimation, thereby lowering the performance of blood pressure measurement based on BCG. In this study, to prevent such performance degradation, a binary classification model was created to distinguish between high-quality versus low-quality BCG signals. To estimate the most accurate model, four time-series imaging methods (recurrence plot, the Gramain angular summation field, the Gramain angular difference field, and the Markov transition field) were studied to convert the temporal BCG signal associated with each heartbeat into a 448 × 448 pixel image, and the image was classified using CNN models such as ResNet, SqueezeNet, DenseNet, and LeNet. A total of 9626 BCG beats were used for training, validation, and testing. The experimental results showed that the ResNet and SqueezeNet models with the Gramain angular difference field method achieved a binary classification accuracy of up to 87.5%.

## 1. Introduction

Blood pressure (BP) measurement provides useful information about the state of the heart and physiological changes in the body. The presence or progression of diseases can be monitored through information on blood pressure changes [1,2]. In general, the non-invasive BP measurement methods most commonly used in hospitals and homes are the auscultatory [3] and oscillometric [4] methods. These methods commonly use a cuff to put blood vessels in a compression–relaxation state by inflating and deflating the cuff. The oscillometric method is widely used for automatic BP measurement based on the size and shape of the pulsation or oscillation of blood vessels during the cuff deflation process (called an “oscillogram”) [5]. The other method, auscultation, is the gold standard for clinical BP measurement [6] because of its reliable clinical history. In the auscultatory method, BP is measured by a trained expert by detecting Korotkoff sounds through a stethoscope and a sphygmomanometer while deflating a cuff. A key limitation of these methods is that they are not suited to continuous and ubiquitous BP measurement for various healthcare applications.

Ubiquitous BP monitoring has the potential to prevent and manage cardiovascular diseases in advance. In the case of hypertension, it is important for patients to try to control their own BP by frequently monitoring it and taking appropriate anti-hypertensive medications. Furthermore, in a situation of measuring BP at a medical institution or hospital, a white-coat effect may occur, in which BP rises due to conscious or unconscious anxiety in a patient. This results in a higher BP measurement than the actual BP of a patient. To prevent this bias error, it is vital to obtain the average BP through continuous measurements and find the right trend of BP fluctuations in daily life by excluding the instantaneous random effects of psychological and physical states. According to the 2018 European Society of Cardiology/European Society of Hypertension (ESC/ESH) guideline for treating hypertension [7], continuous BP measurement for at least 3 consecutive days is recommended to provide care for hypertension.

With the need for continuous and ubiquitous BP measurement, the method of cuff-less BP measurement with wearable devices has been widely studied in the literature. One approach is to find a representative feature that has a strong correlation with BP changes. One representative feature that has been actively studied is the pulse transit time (PTT), which is inversely proportional to BP [8]. The PTT is the travel time of the arterial pulse pressure wave from a proximal point from the heart to a distal point. To calculate the PTT, we need two distinct bio-signal sources for proximal and distal time events; for example, one should synchronize with the start of a heartbeat, while the other should detect a timed event of the propagation of the BP wave to a distal point. Because of the two distinct sources that need to be measured, the PTT is not easily determined in a wearable device set. Another feature is the pulse arrival time (PAT), which is calculated as an interval from the R wave of ECG to the timing of the pulse wave at a distal point. The timing of the pulse wave at a distal point is conveniently measured using photoplethysmogram (PPG) signals [9]. However, the PAT includes the pre-ejection period (PEP), which is the period during the isovolumic phase of the heart and is not related to the progression of the BP wave; there is a gap between the beginning of ventricular depolarization monitored via ECG and the start of blood ejection from the aortic valve. Hence, this time gap causes an error source in estimating BP via the PAT.

In the existing body of the literature on cuff-less BP measurement, the use of a ballistocardiogram (BCG) has been studied as a proximal signal source, which includes a fiducial point that closely corresponds to blood ejection by the heart (i.e., the opening of the aortic valve) to approximate the PTT. A BCG is a ballistic physical signal produced by each heartbeat and is easily measured using an accelerometer. This is a strong advantage of BCG-based PTT measurement for wearable BP-monitoring applications. Despite the potential of BCG, there exists a limitation against the practical use of BCG-based wearable BP measurement; in general, a wearable BCG can be measured using accelerometers, and the magnitude of BCG is a few tens of milli-gravity (one gravity is about 9.8 m/s^2^), which means that BCG is highly prone to motion artifacts and has a low signal-to-noise ratio.

A possible strategy to overcome these limitations is to find high-quality BCG beats suited to BP measurement during signal acquisition and infer BP based only on these high-quality BCG beats. Every heartbeat generates a corresponding BCG signal, which means that about 60 to 100 BCG signals are generated every minute. If there are at least a small number of high-quality BCG beats, they can be readily used to monitor BP trends continuously. Based on this perspective, this study focused on developing a method for assessing the quality of BCG signals that can classify high-quality BCG beats against low-quality BCG beats.

The main contribution of this study is to propose a methodology to distinguish high-quality BCG beats from acquired limb BCG signals that has the potential to be used for physiological monitoring via wearable devices. When selectively using high-quality BCG beats and ignoring low-quality ones for physiological analysis, the reliability and accuracy of BCG-based physiological monitoring are likely to be improved. Since a BCG beat is a series of data points ordered by time, known as a time series, differentiating high-quality and low-quality BCG beats can be considered a time-series classification problem. In this study, we applied time-series imaging methods, including a recurrence plot [10], a Gramian angular field, and a Markov transition field [11] to expand the feature space of the BCG signal from a 1D time series to 2D images, which may open an opportunity to extract more informative features for the time-series classification. After transforming time-series data into an image, this classification problem can be converted into an image classification problem, which has been widely studied in the literature. To tackle the image classification problem, we adopted a convolutional neural network (CNN) because a CNN can effectively capture local features underlying complex images and incorporate the key features. The characteristics of a CNN dramatically increase the recognition capability for complex images. Based on these benefits, we used the CNN-based classification algorithms LeNet [12], ResNet [13], DenseNet [14], and SqueezeNet [15] for distinguishing the converted BCG time-series images into high-quality versus low-quality ones. To demonstrate the effectiveness of the proposed approach, we used a CNN proposed by Wang [16] for 1D time-series classification as the baseline.

## 2. Related Works

The related studies on signal quality assessment using BCG are as follows. Zeng [17] proposed a 1D CNN to classify cardiac abnormality. The collected signals were whole-body BCG signals measured from a chair using a polyvinylidene fluoride piezoelectric film sensor. The proposed 1D CNN classification model consisted of two 1D convolutional layers, an average pooling layer, and a max pooling layer. The dataset used contained four types of BCG signals: healthy young, healthy elderly, elderly with cardiac abnormalities, and elderly with heart disease. The authors obtained an accuracy of >91% in their results in their multi-class classification.

Hong [18] developed a chair system that could measure bio-signals (ECG, PPG, and BCG) for smart healthcare applications. The authors evaluated the signal quality of the measured signals to confirm the suitability of the multimodal bio-signal chair system for daily life data collection. The collected signal morphology was distorted by motion artifacts, and the materials between the skin and sensors further deteriorated the signal transmission conditions. To quantify this distortion, the authors proposed a signal quality index, which was defined by the similarity between the observed signals and the pre-determined noise-free template signal and determined the effect of the various motions on the signal reliability. However, the naïve morphological similarity with the reference used in the work could be less informative if the timing of specific peak events in the bio-signals were to be estimated as representative features.

Wang [19] proposed a classification method to categorize four types of BCG signals: healthy young people, healthy elderly people, abnormal human bodies, and human heart disease. The proposed method was developed by determining shapelet features, which are subsequences of the time series, to differentiate different time series. The authors studied an enhanced self-organizing incremental unsupervised neural network to obtain a candidate set of shapelet features. Subsequently, the candidate set was converted to the shapelet space. Feature selection and model training were performed in the shapelet space. The authors used an L_2_ regularization-based embedded method for feature selection and the one-vs-all-strategy-based SVM for classification. The proposed technique, shapelet-based BCG classification, has the ability to classify both high- and low-quality BCG signals.

Bicen [20] proposed a BCG signal quality index to assess the effect of noise on the BCG signal. The authors calculated the signal quality index as the average correlation coefficient between sub-templates and parent templates. The sub-templates and parent templates were subject-specifically estimated by averaging the BCG beats segmented using the ECG R waves. The difference between the sub-template and parent template was that the former was calculated using a subset of the BCG beats and the latter was determined using all the recorded BCG beats. The noise statistics were calculated from the sub-templates for the parent template. Based on the knowledge from the noise statistics, the authors analyzed the impact of additive noise on the BCG morphology and also estimated the physiological parameters.

In the literature, signal quality assessment with ECG [21,22,23,24,25] and PPG [26,27,28,29,30,31] was actively studied owing to the popularity of these signals in healthcare and medical applications. Compared to them, the related works on BCG signal quality assessment were relatively fewer than those on PPG and ECG signals. Moreover, the work was limited to the whole-body BCG signal, which represents the movements of a body along the gravitational axis. Although the arm BCG signal is more practical than the whole-body BCG signal as a wearable device for BP measurement, studies on arm BCG signal quality assessment were difficult to find in the literature. Owing to this limitation, this study proposed a methodology for arm BCG signal quality assessment using time-series imaging methods and CNN-based classifications.

## 3. Materials and Methods

### 3.1. BCG Dataset [8]

We used experimental data collected from previous work [8]. The dataset included ECG, PPG, and BCG data under the approval of the Institutional Review Board (IRB) at the University of Maryland and with written informed consent. The acquired bio-signals included: (i) ECG signals measured using three-gel electrodes in the Lead II configuration (BN-EL50, Biopac Systems, Goleta, CA, USA), (ii) a finger clip PPG (TSD124A, Biopac Systems, Goleta, CA, USA), and (iii) an arm BCG measured using a three-axis accelerometer (BN-ACCL3, Biopac Systems, Goleta, CA, USA). All the signals were recorded using a data acquisition unit (MP150 Biopac Systems, Goleta, CA, USA) at a 1 kHz sampling rate.

**Figure 1:**
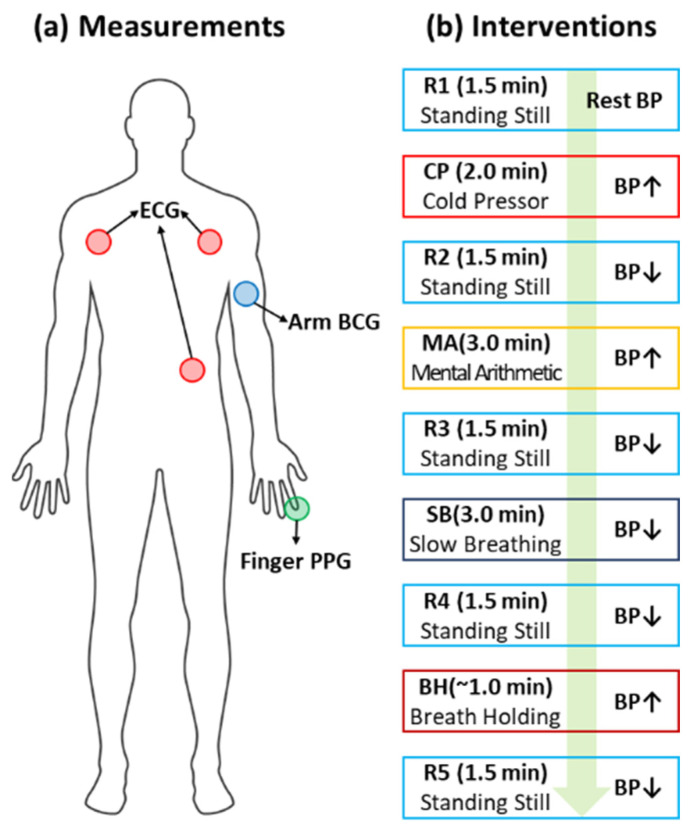
BCG dataset. (**a**) Bio-signals used in the study. (**b**) Interventions performed to perturb blood pressure. The green arrow indicates the procedure of the interventions.

The experimental data were collected while the participants underwent four BP-perturbing interventions: cold pressor (CP), mental arithmetic (MA), slow breathing (SB), and breath holding (BH). Every intervention was followed by standing still for 90 s for the resting state. First, the participants undertook the CP intervention for up to 120 s, in which they were asked to immerse their free hand in ice water. Second, the participants undertook the MA intervention for up to 180 s, in which they were asked to repeatedly add the digits of a three-digit number and add the sum to the original number. Third, the participants went through the SB intervention for up to 180 s, in which they were asked to repeatedly take slow and deep breaths. Fourth, the participants performed the BH intervention, in which they were asked to hold their breath after normal exhalation. During the data acquisition, the participants were asked to stand still and minimize the movements of their arms by placing them on their side. Figure 1 shows the types and locations of the biosensors and the interventions the participants underwent during the data acquisition.

**Figure 2:**
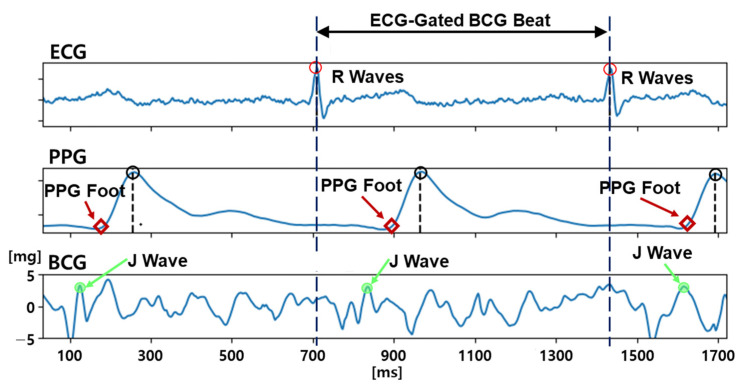
ECG, PPG, and BCG after pre-processing and segmentation procedures.

To pre-process the data, we focused on the acceleration in the head-to-foot direction as the wearable BCG signal. Filtering (ECG pass band: 0.5–100 Hz, PPG and BCG pass band: 0.5–20 Hz) and beat segmentation by the ECG R waves were carried out using Scipy and Neurokit2 [32]. First, the ECG R waves were detected by a derivative (slope of the tangent) and a waveform height threshold of the ECG signal, and the R waves were used to gate the heartbeats. In each beat, the PPG foot was detected using the intersecting tangent method [33]. Finally, the J-wave candidate point in the BCG, which is the vital marker to determine the quality of the BCG, was detected visually in each heartbeat. Figure 2 shows the pre-processed and segmented ECG, PPG, and BCG signals.

### 3.2. BCG Quality Labeling Procedure

**Figure 3:**
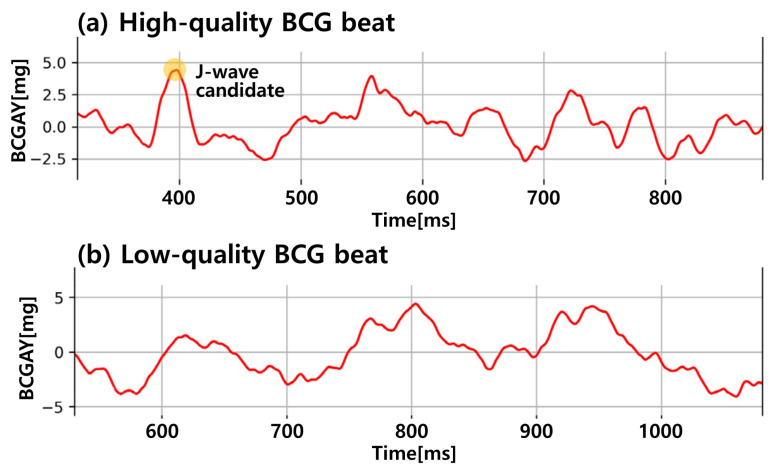
Examples of high- and low-quality BCG beats.

In terms of the quality of the BCG beats, we introduced two types: high- and low-quality BCG beats, as shown in Figure 3. These types were discriminated by the following rules:

(1) The existence of a distinguishable and sharp J-wave candidate before the PPG foot,
(2) The magnitude of the J-wave candidate being more than 3 mg (micro-gravity), and
(3) The width of the J-wave candidate being less than 100 ms.

If a BCG beat satisfied all the rules, it was labeled as high-quality; if not, the beat was labeled low-quality. This labeling procedure was performed by the visual inspection of a trained expert. After completing the labeling task, the number of low-quality beats was relatively higher than their high-quality counterparts because the signal-to-noise ratio of the BCG signals is typically low, which means that it is easy to collect low-quality BCG beats and laborious to gather high-quality BCG beats. To avoid the data imbalance problem, we selected the same number of high- and low-quality BCG beats (4813 samples were chosen for the analysis, respectively). Figure 3 shows two examples of high- and low-quality BCG beats. This labeling procedure was performed using the BCG signals of 8 subjects from the previously collected dataset [8].

### 3.3. Time-Series Imaging Methods

#### 3.3.1. Recurrence Plot

The recurrence plot (RP) is a time-series data visualization method to represent the recurring nature of states for each moment *i* in time [10]. The basic formulation of the RP is as follows. Given a time series $\left(x_{1},\;\cdots ,\;x_{n}\right)$ with a length *n*, the trajectory at a discrete time point *i* is as follows:

(1) $${\vec{x}}_{i}\;=\left(x_{i},\;x_{i+\tau },\;\cdots ,\;x_{i+\left(m-1\right)\tau }\right),\;\;\forall i\in \left\{1,\ldots ,n-\left(m-1\right)\tau \right\}$$

where *m* is the dimension of the trajectories and τ is the time delay. With these trajectories, the binarized recurrence matrix ${\hat{R}}_{i,j}$ with *i*, *j* discrete time points is the pairwise distance between the trajectories and is shown as follows:

(2) $${\hat{R}}_{i,j}=\Theta \left(\varepsilon -\left\|{\vec{x}}_{i}-{\vec{x}}_{j}\right\|\right),\;\;\forall i,j\in \left\{1,\ldots ,n-\left(m-1\right)\tau \right\}$$

where $\left\|\cdot \right\|$ is a norm operation, Θ is the Heaviside (step) function, and ε is the recurrence threshold. As a variation of the above form, only the norm operation can be used to calculate the continuous values of the recurrence matrix. This variation generates a continuous recurrence matrix for the RP expressed by:

(3) $$R_{i,j}=\left\|{\vec{x}}_{i}-{\vec{x}}_{j}\right\|,\;\;\forall i,j\in \left\{1,\ldots ,n-\left(m-1\right)\tau \right\}$$

In this study, we used the continuous recurrence matrix and set the dimension of the trajectories *m* as 1 and the time delay τ as 1 to simplify the calculation. Consequently, the trajectory at the time *i* had only one element of the given time series at the time *i*, ${\vec{x}}_{i}\;=\left(x_{i}\right)$.

#### 3.3.2. Gramian Angular Field

The Gramian angular field (GAF) [11] is also a time-series data visualization method used to represent a 1D time series in a 2D polar coordinate system. Given a time series $X=\left(x_{1},\;\cdots ,\;x_{n}\right)$ of *n* observations, the general procedure of the GAF is as follows.

First step: apply the min–max normalization to X so that all observations fall into the interval [−1, 1]:

(4) $$\tilde{X}=\left({\tilde{x}}_{1},\;\cdots ,\;{\tilde{x}}_{n}\right),\;\;{\tilde{x}}_{i}=\frac{\left(x_{i}-\max\left(X\right)+\left(x_{i}-\min\left(X\right)\right)\right)}{\max\left(X\right)-\min\left(X\right)}$$

Second step: project the rescaled time series $\tilde{X}$ in the polar coordinates by converting the rescaled observations ${\tilde{x}}_{i}$ as the angle $\phi_{i}$:

(5) $$\Phi =\left(\phi_{1},\;\cdots ,\;\phi_{n}\right),\;\;\phi_{i}=\cos^{-1}\left({\tilde{x}}_{i}\right),\;-1\;\leq {\tilde{x}}_{i}\;\leq 1,\;{\tilde{x}}_{i}\in \tilde{X}$$

Optionally, there is a variation to differentiate the number of observations in the time series, *n* in ${\tilde{x}}_{n}$, and the size of the output image, *n* in $\phi_{n}$ by applying the piecewise aggregate approximation (PAA) [34] method to the time series before the projection.

Third step: generate a GAF matrix by the angle $\phi_{i}$:

There are two approaches to generate the GAF matrix: (i) the Gramian angular summation field (GASF) using the trigonometric sum with the cosine function and (ii) the Gramian angular difference field (GADF) using the trigonometric difference with the sine function:

(6) $$GASF=\left[\begin{array}{ccc} \cos\left(\phi_{1}+\phi_{1}\right) & \cdots & \cos\left(\phi_{1}+\phi_{n}\right)\\ \cos\left(\phi_{2}+\phi_{1}\right) & \cdots & \cos\left(\phi_{2}+\phi_{n}\right)\\ \vdots & \ddots & \vdots \\ \cos\left(\phi_{n}+\phi_{1}\right) & \cdots & \cos\left(\phi_{n}+\phi_{n}\right) \end{array}\right]={\tilde{X}}^{T}\cdot \tilde{X}-{\sqrt{I-{\tilde{X}}^{2}}}^{T}\cdot \sqrt{I-{\tilde{X}}^{2}}$$

(7) $$GADF=\left[\begin{array}{ccc} \sin\left(\phi_{1}-\phi_{1}\right) & \cdots & \sin\left(\phi_{1}-\phi_{n}\right)\\ \sin\left(\phi_{2}-\phi_{1}\right) & \cdots & \sin\left(\phi_{2}-\phi_{n}\right)\\ \vdots & \ddots & \vdots \\ \sin\left(\phi_{n}-\phi_{1}\right) & \cdots & \sin\left(\phi_{n}-\phi_{n}\right) \end{array}\right]={\sqrt{I-{\tilde{X}}^{2}}}^{T}\cdot \tilde{X}-{\tilde{X}}^{T}\cdot \sqrt{I-{\tilde{X}}^{2}}$$

where *I* is the unit row vector [1, 1, …, 1] of dimension *n* and ${\tilde{X}}^{2}$ is the element-wise square.

#### 3.3.3. Markov Transition Field

Another time-series imaging method is the Markov transition field (MTF) [11], which uses the concept of the Markov transition probability to encode the dynamical transition statistics of the time-series data while preserving temporal information. Given a time series $X=\left(x_{1},\;\cdots ,\;x_{n}\right)$, we configure *Q* quantile bins and allocate each observation $x_{i}$ to the corresponding bin $q_{j}$ (j∈[1,Q]) so that each quantile bin has the same number of observations. We then calculate a *Q* × *Q* Markov transition matrix *W*, which is a weighted adjacency matrix, by calculating the first-order Markov chain transitions of the quantile bins:

(8) $$W\;=\left[\begin{array}{ccc} w_{11} & \cdots & w_{1Q}\\ \vdots & \ddots & \vdots \\ w_{Q1} & \cdots & w_{QQ} \end{array}\right],\;\;w_{ij}=\sum_{k=1}^{n-1}\left[x_{k}\in q_{i}\;AND\;x_{k+1}\in q_{j}\right]$$

Here, […] is the Iverson brackets, and $w_{i,j}$ is determined by the frequency with which a point in the quantile $q_{j}$ is followed by a point in the quantile $q_{i}$. After calculating the Markov transition matrix *W*, column-wise normalization is performed on the elements of the matrix as follows:

(9) $${\hat{w}}_{ij}=\frac{w_{ij}}{\sum_{j}w_{ij}}$$

However, the Markov transition matrix *W* has no temporal information nor a distribution of X. To overcome this information loss, the MTF matrix *M* is defined as

(10) $$M=\left[\begin{array}{ccc} {\hat{w}}_{ij\;|x_{1}\in q_{i},x_{1}\in q_{j}} & \cdots & {\hat{w}}_{ij\;|x_{1}\in q_{i},x_{n}\in q_{j}}\\ {\hat{w}}_{ij\;|x_{2}\in q_{i},x_{1}\in q_{j}} & \cdots & {\hat{w}}_{ij\;|x_{2}\in q_{i},x_{n}\in q_{j}}\\ \vdots & \ddots & \vdots \\ {\hat{w}}_{ij\;|x_{n}\in q_{i},x_{1}\in q_{j}} & \cdots & {\hat{w}}_{ij\;|x_{n}\in q_{i},x_{n}\in q_{j}} \end{array}\right]$$

$M_{ij}$ represents the transition probability of $q_{i}\to q_{j},\forall i,j\in [1,Q]$. That is, the Markov transition matrix only indicates the transition probability on the magnitude axis. However, the MTF matrix denotes the spread of the transition probability along the temporal axis, which means the matrix includes the temporal information of X.

**Figure 4:**
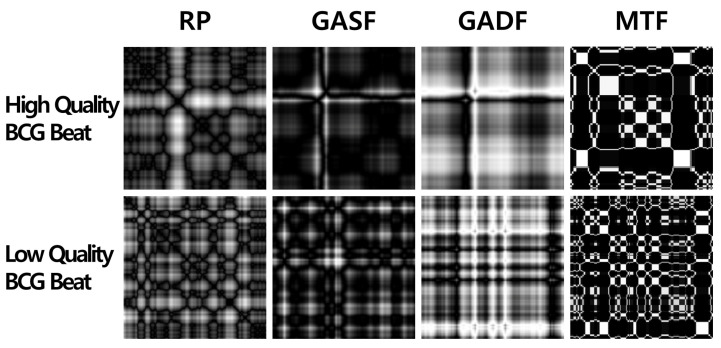
Examples of imaging a BCG time-series beat by recurrence plot (RP), Gramian angular summation field (GASF), Gramian angular difference field (GADF), and Markov transition field (MTF).

Figure 4 shows examples of imaging high- and low-quality BCG beats via RP, GASF, GADF, and MTF.

#### 3.3.4. CNN-Based Binary Classification for BCG Signal Quality Assessment

This study used a total of four methods to image the time series of the BCG beats: RP, GASF, GADF, and MTF. From these methods, each BCG beat was converted to 2D gray-scale images. To determine the BCG signal quality, five 2D CNN-based classification models were considered: ResNet [13], SqueezeNet [15], DenseNet [14], LeNet-Tanh, and LeNet-ReLU. The size of the input image was 448 × 448 pixels for the 2D CNN-based classifiers. The 448 × 448 pixel resolution indicates that the time-series image includes the information of the first 448 ms period of each BCG beat (at 1 kHz sampling rate). The number, 448, was chosen by a factor of the input size of the CNN models used in this study. Moreover, based on our previous study [35], the first 448 ms period data are enough to catch the fiducial point, the J-wave, of a BCG beat. Additionally, a 1D CNN-based classification model, a fully convolutional neural network (FCN) [16], was chosen as a baseline time-series classifier to compare with the performance of the time-series image-based 2D CNN classifications.

LeNet [12] (referred to as LeNet-5) is one of the earliest CNN models that was introduced by LeCun in 1998. LeNet was developed to identify handwritten zip code numbers. Each handwritten 1-digit number was a grayscale image that had a 32 × 32-pixel size. Specifically, LeNet-5 consisted of 7 layers: 3 convolutional layers with 5 × 5 filter size and 1-stride, 2 average pooling layers with 2-stride, and 2 fully connected layers. They used *tanh* functions as the activation functions and the last fully connected layer used a softmax function to calculate the probability of each class for the classification.

We modified the basic LeNet-5 to accept input images with a 448 × 448-pixel size. The modified LeNet (hereinafter referred to as “LeNet-Tanh”) has the additional 2 convolutional layers (with a 5 × 5 filter size and 2-stride) and 2 average-pooling layers (with 2-stride) in order to work properly with the increased input image resolution. Another modified version of LeNet used in the study was “LeNet-ReLU”. The basic LeNet-5 and LeNet-tanh used the tanh activation function; however, LeNet-ReLU used the rectified linear unit (ReLU) activation function instead of the tanh activation function and the max-pooling layers rather than the average-pooling layers.

The residual neural network (ResNet) [13] introduced the residual block, which creates a shortcut between the input layer and output layers to enable the training of deeper neural networks without the vanishing/exploding gradient problems. This “skip connection” (or shortcut connection) between the input and output layers helps to propagate gradients through the neural network in the training phase. The skip connection in ResNet is implemented by summing up the output of a general neural network and the input. In this study, we used a ResNet model with 18 layers.

A dense convolutional network (DenseNet) [14] was introduced to improve the performance with a smaller number of parameters compared to ResNet. The key concept of DenseNet for performance improvement is to use all the previous feature maps for identity mapping. The authors focused on the possibility that the simple summation used in the residual block prevents the information flow in the network. To solve this limitation, they introduced dense connectivity to concatenate all the preceding feature maps for the identity mapping.

DenseNet avoids the vanishing gradient problems by connecting the preceding feature maps, thereby making it possible to expect the effect of feature reuse in the sense that the information from the earlier layers remains alive at the end of the network with dense connectivity. Because of this advantageous aspect, DenseNet outperformed ResNet with a smaller number of parameters and computational burdens.

In the analysis, we used a DenseNet-121 model equipped with four dense blocks, each of which had 6, 12, 24, and 16 channels, respectively.

The goal of SqueezeNet [15] is to create a smaller neural network with fewer parameters while maintaining adequate performance for easy application in harsh computational environments. In [15], the authors stated that AlexNet had 240 megabytes (MB) of parameters and SqueezeNet had only 4.8 MB. To achieve this, the authors proposed three strategies:

(1) Use 1 × 1 filters instead of 3 × 3 filters for fewer parameters.
(2) Decrease the number of input channels to reduce the number of parameters.
(3) Conduct down-sampling at the latter part of the network to gain large activation maps (maximizing the performance with a reduced number of parameters).

To realize these strategies, SqueezeNet included the fire module, which comprises a squeeze layer with 1 × 1 filters (“s1 × 1”) and an expanded layer with a bunch of 1 × 1 and 3 × 3 filters (“e1 × 1”, “e3 × 3”). The authors set the number of hyperparameters in the squeeze layer, s1 × 1, to be smaller than the number of hyperparameters in the expanding layer, e1 × 1 and e3 × 3. This satisfied the second strategy; the squeeze layer helps to limit the number of input channels to the 3 × 3 filters of the expanding layer.

Mainly, SqueezeNet included eight fire modules and had three architectures based on the topology of the bypass connections: SqueezeNet, SqueezeNet with a simple bypass, and SqueezeNet with a complex bypass. Briefly, the concept of the bypass connection is similar to the skip connection in ResNet. The main difference between the simple and complex bypasses is that the complex bypass has a 1 × 1 Conv to match the dimensions of the input and output. For the implementation of SqueezeNet in the BCG signal quality classification, we used a SqueezeNet v1.0 model.

Wang [16] introduced the FCN as a viable baseline method for time-series classification based on deep neural network architectures (1D convolutional layers, batch normalization layers, and ReLU activation layers). In the FCN model, three 1D convolutional layers, which have the different 1D kernel sizes of {8, 5, 3} with no strides and the different filter sizes of {128, 256, 128}, were stacked. Each convolutional layer was followed by a batch normalization layer and a ReLU activation layer in succession. The latter part of the FCN model consisted of a global average pooling layer and a softmax layer. The global average pooling layer was chosen instead of a fully connected layer to reduce the number of parameters.

## 4. Results

The accuracy of the binary classification was evaluated for the performance comparison of five (5) 2D CNN image classifiers each based on four (4) time-series imaging methods as well as the baseline 1D CNN classifier. Stratified 5-fold cross-validation was employed to calculate the standard deviation. We split the dataset (9626 samples) into the training (50%, 4812 samples), validation (20%, 1926 samples), and test (30%, 2888) sets. Moreover, to optimize the hyperparameters, the batch size (64) and epoch (500) were fixed, but the optimizers (Adam, SGD, and RMSprop) and learning rates (from 0.1 to 0.0001) were tuned for each CNN model. Model training and analysis were conducted in Intel i7-11700k, NADIA GeForce RTX 3080, 32 GB RAM with Python (3.9), Pytorch (1.10), Pyts (0.12), and Torchvision (0.11.1).

Table 1 lists the accuracy results with the optimized hyperparameters. A summary of the findings is given below.

- SqueezeNet with GADF resulted in the highest accuracy (87.5%); however, there was no statistically significant difference (*p* < 0.05) with the independent samples *t*-test (*p*-value = 0.64).
- Relatively, the GADF imaging approach outperformed the others in all the 2D CNN classifiers.
- RP and GASF showed similar performance in all the 2D CNN classifiers.
- MTF produced the lowest accuracy.
- The variance of the accuracy alongside the same imaging approach was less than the variance alongside the same 2D CNN classifier.
- The accuracy of all 2D CNN classifiers, except LeNet_Tanh and SqueezeNet with MTF, exceeded that of the 1D CNN approach (baseline).

**Figure 5:**
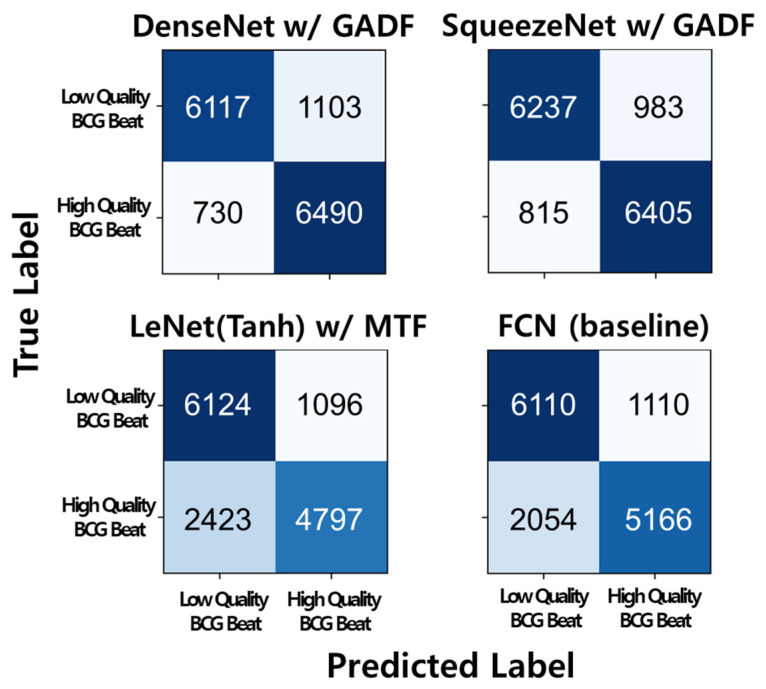
Confusion matrices of SqueezeNet with GADF (87.5% in accuracy), DenseNet with GADF (87.3%), LeNet_Tanh with MTF (75.6%), and the baseline model, FCN, (78.1%). Each experiment had 2888 samples for the test set, and the experiment was repeated five times by five-fold CV. In total, 14,440 samples were used for the confusion matrices. The number in the confusion matrices is the number of samples.

Figure 5 shows the confusion matrices of two of the highest-accuracy results (SqueezeNet and DenseNet with GADF), the lowest-accuracy result (LeNet_Tanh with MTF), and the baseline. From Figure 5, we speculated that the performance drop in the accuracy was due to the misclassification of the high-quality BCG beat. The two confusion matrices with the highest accuracy showed that the misclassification of the high-quality BCG beat included 730 samples in DenseNet with GADF and 815 samples in SqueezeNet with GADF. However, the confusion matrices of the lowest accuracy and baseline showed that the misclassification of the high-quality BCG beat amounted to 2423 samples in LeNet_Tanh with MTF and 2054 samples in FCN. The misclassifications of the low-quality BCG beat of the four confusion matrices were slightly different; however, in terms of the magnitude of their difference, they seemed less significant.

**Figure 6:**
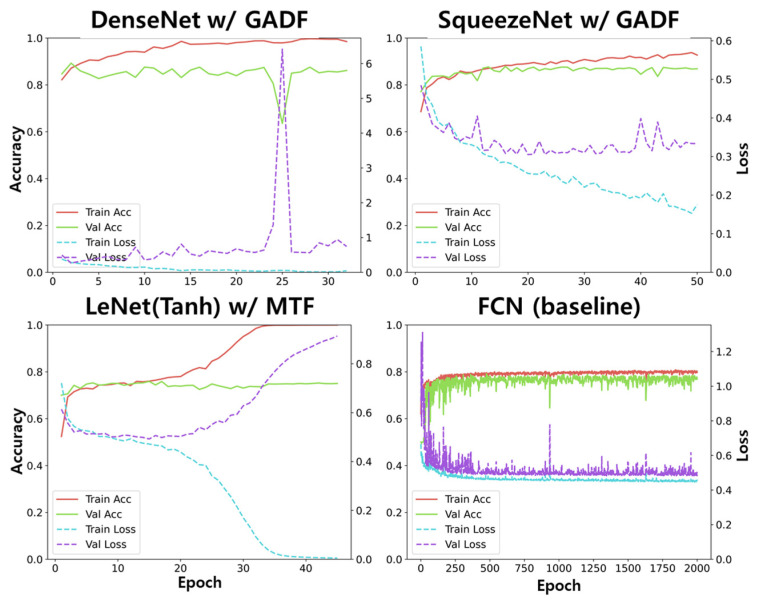
Plots of training/validation accuracy, [0, 1], and loss, [0, ~), of SqueezeNet with GADF (87.5% in accuracy), DenseNet with GADF (87.3%), LeNet_Tanh with MTF (75.6%), and the baseline model, FCN, (78.1%).

Figure 6 shows the plots of the accuracy and loss in the training and validation procedures of SqueezeNet with GADF, DenseNet with GADF, LeNet_Tanh with MTF, and FCN. Until the saturation of the validation accuracy, the 2D CNN models needed less than 10 epochs. However, the 1D CNN model, FCN, needed approximately 250 epochs in the training procedure. From the results, the 1D CNN model needed more training epochs than the 2D CNN model. Although the number of epochs in training was not directly correlated to the computational time, the 1D CNN model needed more repeated computations to acquire a trained model.

## 5. Discussion and Conclusions

This work evaluated the arm BCG signal quality classification problem using time-series imaging approaches. The arm BCG signal is a bio-signal, which has the potential to enable cuff-less BP estimation. A well-known challenge associated with the arm BCG signal is its low signal-to-noise ratio. Although the weakness still exists, there is a possibility to overcome this weakness for the practical use of the arm BCG signal for BP measurement in daily life applications. To realize this possibility, we introduced a methodology to determine high-quality BCG beats using time-series imaging approaches. The proposed approaches outperformed the 1D CNN-based classifier in the BCG signal quality assessment. The 2D CNN classifier based on SqueezeNet, which converts 1D time-series BCG beats into 2D images using the Gramian angular difference field, had an 87.5% accuracy. The improved performance may be attributed to the ability of the time-series imaging method to expand the feature space. Moreover, in terms of the image classification, we successfully took advantage of the well-developed results from the image classification literature. Moreover, in terms of the training epoch, we deduced that the 2D CNN models needed a few epochs for training compared to the 1D CNN model. This is not the absolute measure to estimate the computational time and burdens for training a model. However, we speculated that the 2D CNN models with the time-series images were quickly trained to discriminate between the high- and low-quality BCG beats compared to the 1D CNN model.

This study has a few limitations. First, the real-time performance of the proposed approach for practical uses was not studied. In the proposed approach, the computational burden could be increased by the time-series imaging procedure. However, the computational burden, caused by performing the time-series imaging procedure and the architectural difference between the 2D and 1D CNN models, was not evaluated in this study because the main goal was to determine the attainable potentials of the BCG classification methods through a transformation of the time series into imaging. Although the level of real-time capability for healthcare applications may not be as strict as industrial mission-critical real-time systems, it is necessary to examine the real-time feasibility of the proposed approach in follow-up work.

Second, the dataset used in the study was not enough to represent inter-subject variability. Although the size of the dataset was relatively enough (9626 BCG beats in total), it was only collected from eight healthy human subjects. In terms of the condition and size of the subjects, the morphological variation in the BCG signal was limited. Subsequent work needs to increase the number of recruited human subjects with regard to age, sex, race, etc. Considering the listed limitations is essential for BP measurement in wearable systems.

In summary, this study showed the possibility of using time-series imaging methods for BCG signal quality classification. This approach is advantageous in terms of (i) increasing the dimensions of the time-series information and (ii) leveraging the mature CNN architecture for the classification. Future work should consider developing a computationally efficient time-series imaging method and testing the reliability of the signal quality assessment algorithm in an embedded system.

## Figures and Tables

**Table 1:** Accuracy of CNN-based classifiers (mean ± SD).

		2D CNN-Based Classifier					(Baseline) *
		ResNet	SqueezeNet	DenseNet	LeNet (Tanh)	LeNet (ReLU)	FCN
Time-series Image	RP	83.1%	82.9%	84.0%	82.9%	83.7%	78.1% (±1.5%)
		(±1.4%)	(±1.3%)	(±0.4%)	(±0.6%)	(±0.6%)	
	GASF	84.1%	82.3%	84.0%	81.6%	83.3%	
		(±0.7%)	(±0.7%)	(±0.8%)	(±0.9%)	(±0.6%)	
	GADF	86.7%	**87.5%**	87.3%	85.6%	86.9%	
		(±0.9%)	(±0.7%)	(±0.9%)	(±0.5%)	(±0.9%)	
	MTF	79.6%	76.7%	78.5%	75.6%	78.8%	
		(±1.2%)	(±1.4%)	(±1.4%)	(±0.4%)	(±0.6%)	

* One-dimensional CNN-based classifier.

## Data Availability

The data presented in this study are available on request from the corresponding author.
